# Kawasaki disease, multisystem inflammatory syndrome in children, and adenoviral infection: a scoring system to guide differential diagnosis

**DOI:** 10.1007/s00431-023-05142-6

**Published:** 2023-08-19

**Authors:** Marianna Fabi, Arianna Dondi, Laura Andreozzi, Leonardo Frazzoni, Giovanni Battista Biserni, Francesco Ghiazza, Elton Dajti, Rocco Maurizio Zagari, Marcello Lanari

**Affiliations:** 1 Pediatric Emergency Unit, IRCCS Azienda Ospedaliero-Universitaria di Bologna, via Massarenti 9, 40138, Bologna, Italy; 2 Department of Medical and Surgical Sciences, Alma Mater Studiorum, University of Bologna, Bologna, Italy; 3 IRCCS Azienda Ospedaliero-Universitaria di Bologna, Bologna, Italy; 4 Department of Medical and Surgical Sciences, University of Bologna, Bologna, Italy; 5 Specialty School of Pediatrics, Alma Mater Studiorum, University of Bologna, Bologna, Italy; 6 Alma Mater Studiorum, University of Bologna, 40126 Bologna, Italy

**Keywords:** Kawasaki disease, MIS-C, Adenovirus, Viral infections, Fever, Diagnostic score

## Abstract

Children with Kawasaki disease (KD), Multisystem Inflammatory Syndrome in Children (MIS-C), and Adenovirus infections (AI) of the upper respiratory tract show overlapping features. This study aims to develop a scoring system based on clinical or laboratory parameters to differentiate KD or MIS-C from AI patients. Ninety pediatric patients diagnosed with KD (n = 30), MIS-C (n = 26), and AI (n = 34) admitted to the Pediatric Emergency Unit of S.Orsola University Hospital in Bologna, Italy, from April 2018 to December 2021 were enrolled. Demographic, clinical, and laboratory data were recorded. A multivariable logistic regression analysis was performed, and a scoring system was subsequently developed. A simple model (clinical score), including five clinical parameters, and a complex model (clinic-lab score), resulting from the addition of one laboratory parameter, were developed and yielded 100% sensitivity and 80% specificity with a score ≥2 and 98.3% sensitivity and 83.3% specificity with a score ≥3, respectively, for MIS-C and KD diagnosis, as compared to AI.

*Conclusion*: This scoring system, intended for both outpatients and inpatients, might limit overtesting, contribute to a more effective use of resources, and help the clinician not underestimate the true risk of KD or MIS-C among patients with an incidental Adenovirus detection.

**Table:** 

**What is Known:** *• Kawasaki Disease (KD), Multisystem Inflammatory Syndrome in Children (MIS-C) and adenoviral infections share overlapping clinical presentation in persistently febrile children, making differential diagnosis challenging.* *• Scoring systems have been developed to identify high-risk KD patients and discriminate KD from MIS-C patients.*	
**What is New:** *• This is the first scoring model based on clinical criteria to distinguish adenoviral infection from KD and MIS-C.* *• The score might be used by general pediatricians before referring febrile children to the emergency department.*

**What is Known:**

*• Kawasaki Disease (KD), Multisystem Inflammatory Syndrome in Children (MIS-C) and adenoviral infections share overlapping clinical presentation in persistently febrile children, making differential diagnosis challenging.*

*• Scoring systems have been developed to identify high-risk KD patients and discriminate KD from MIS-C patients.*

**What is New:**

*• This is the first scoring model based on clinical criteria to distinguish adenoviral infection from KD and MIS-C.*

*• The score might be used by general pediatricians before referring febrile children to the emergency department.*

## Introduction

General pediatricians face a diagnostic dilemma with persistently febrile children who present a miscellaneous of signs of vasculitis, lymphadenopathies, and abdominal manifestations. Kawasaki disease (KD), Multisystem Inflammatory Syndrome in Children (MIS-C), and Adenovirus infections (AI) of the upper respiratory tract are affections that share an aberrant systemic inflammation and overlapping clinical signs and symptoms. Clinical presentations include high-grade fever, multiple lymphadenopathies, cutaneous and mucosal changes, conjunctivitis, and abdominal symptoms. However, disease severity is often different, being usually milder in AI and more serious in KD and MIS-C. Furthermore, these conditions require different management: AI patients only need supportive therapy and can usually be safely discharged home; conversely, hospitalization, close monitoring, and immunomodulatory and antiplatelet agents are recommended for KD and MIS-C patients, for whom careful fluid replacement and anticoagulation can be indicated.

KD is a systemic vasculitis involving medium-sized arteries, notably coronary arteries, and is the leading cause of acquired heart diseases in childhood in high-income countries [1]. Its diagnosis is based on clinical criteria and supported by non-specific laboratory findings of inflammation.

MIS-C, first described after the beginning of the SARS-CoV-2 pandemic [2], is a novel, post-infectious disease developing 2–6 weeks after the SARS-CoV-2 infection and affecting 1–2/200.000 subjects younger than 19 or 21 years, depending on the preferred definition [3, 4]. In addition to an epidemiological link to SARS-CoV-2, the diagnostic criteria overlap with those of KD, including long-lasting fever, skin rash, gastrointestinal complaints, edema of hands and feet, mucosal changes, conjunctivitis, swollen lymph nodes, and laboratory evidence of systemic inflammation.

AI of the respiratory tract can be responsible for a miscellaneous range of high-grade fever, pharyngitis, mucositis, conjunctivitis, cervical lymphadenopathy, gastroenteritis, and erythematous rash, mimicking both KD and MIS-C [5]. Despite being a self-limiting viral infection, the symptoms may persist for several days, and the patient's condition can get worse, increasing the concern of parents and physicians.

The overlapping features of KD, MIS-C, and AI can cause unnecessary hospitalizations and overtesting, with the prescription of blood tests, cardiac evaluations, and microbiological investigations, increasing the burden of outpatient visits and medical expenses. Unfortunately, point-of-care tests for rapid detection of AIs in the field are still mainly used only for research purposes [6, 7].

The aim of the present study is to identify clinical or laboratory features to discriminate between children with KD or MIS-C and those with AI and to develop a practical scoring system accordingly.

## Methods

In this cross-sectional study, all pediatric patients diagnosed with KD, MIS-C, and upper respiratory tract AI admitted to the Pediatric Emergency Unit ward of the Sant’Orsola University Hospital in Bologna, Italy, from April 2018 to December 2021 were enrolled. Due to the association with SARS-CoV-2 infection, patients affected by MIS-C were enrolled from April 2020 (first case diagnosed in our center) to the end of the study.

KD diagnoses were made according to the 2017 American Heart Association (AHA) Guidelines [1]. MIS-C was defined according to the WHO criteria, including clinical, laboratory, and microbiological features, in patients with evidence of SARS-CoV-2 infection or likely contact with confirmed cases [4, 8]. AI was diagnosed by the presence of clinical signs and/or symptoms of upper respiratory tract infection at physical examination, fever >38.5 °C, and at least two of the following: positive naso-pharyngeal swabs for Adenoviral antigen (immunofluorescence), positive IgM on Adenoviral serology (immunoenzymatic assay), or Adenovirus DNA-Polymerase Chain Reaction (PCR, multiplex real-time PCR) on blood or target fluids.

A database was prospectively created and then retrospectively reviewed; it included demographic features (gender, age) and clinical characteristics (non-exudative conjunctivitis, oral mucositis or cheilitis, skin rash, extremity changes, cervical lymphadenopathy, pharyngotonsillitis, rhinitis, cough, bronchitis and/or pneumonia, vomiting, diarrhea, diaper/perineal rash, fever duration), length of hospital stay, and laboratory values of the acute stage of the illnesses (white blood cell count [WBC] with differential, hemoglobin [Hb], platelets [PLT], C-reactive protein [CRP], procalcitonin, serum albumin, aspartate aminotransferase [AST], alanine aminotransferase [ALT], and urine WBC). Gastrointestinal complaints, including vomiting and diarrhea, were registered during the acute stage of the illness using standard definitions, as previously reported [9].

Patients with >5 days of fever, even in the case of AI diagnosis, underwent echocardiography to assess coronary dimensions and systolic function to evaluate the cardiological diagnostic criteria for KD and MIS-C, respectively.

Exclusion criteria included the presence of co-morbidities such as gastrointestinal anatomical malformations, congenital heart disease, chronic or end-stage renal failure, primary immune system deficiency, solid tumors or malignant hematological disease, stem cell transplantation recipients, children on biological and immunomodulating therapies, and patients with infections other than upper respiratory tract AI at admission.

The study was approved by the local Investigational Review Board. Written informed consent was collected by parents/legal tutors for each participant. The study was conducted according to the guidelines of the Declaration of Helsinki and approved by the Ethics Committee of Area Vasta Emilia Centro (approval codes EM 566-2018_340/2017/O/Oss/AOUBO and. 391/2019/Sper/AOUBo; protocol numbers: 98/2016/O/Sper/AOUBo and 178/2021/Sper/AOUBo).

## Statistical analysis

Categorical variables were described as frequencies and percentages, whereas continuous variables were described as the median and interquartile range. Categorical variables were compared by the Chi-square test or Fisher’s exact test, as appropriate, and continuous variables by the Mann-Whitney U test.

A multivariable logistic regression analysis was performed to identify independent predictive factors for MIS-C or KD, using subjects with AI as the baseline group. Odds ratios (ORs) and 95% confidence intervals (CI) were calculated and estimated for the included variables. A predictive model was subsequently developed. A score of 1 point was attributed to each predictor of MIS-C and KD, yielding a total score ranging from 0 to 5 points. The diagnostic accuracy of the model was explored by plotting a receiver operating characteristic (ROC) curve, thereby computing sensitivity, specificity, positive predictive value (PPV), and negative predictive value (NPV) for the absence of risk factors and the presence of each of them. The score maximizing sensitivity was chosen as the best cutoff. A p value < 0.05 was considered statistically significant. Post-hoc power analysis was performed in order to assess the reliability of the logistic regression models. Statistical analyses were performed using STATA version 16 statistical software (Stata Corp., College Station, Texas, USA).

## Results

A total of 90 patients were enrolled: 30 with AI, 26 with MIS-C, and 34 with KD. Demographic and clinical data are reported in Table 1, while laboratory values are shown in Table 2. Only 1 patient met both criteria for AI and KD, but she was diagnosed as KD due to the coronary involvement (right coronary artery dilatation).

**Table 1:** Gender, age, symptoms, and signs of patients with upper respiratory tract Adenovirus infection (AI), multisystem inflammatory syndrome in children (MIS-C), and Kawasaki disease (KD)

	**AI** **n = 30**	**MIS-C** **n = 26**	**p-value** **AI vs MIS-C**	**KD** **n = 34**	**p-value** **AI vs KD**
	**n (%)**	**n (%)**		**n (%)**	
Gender					
Male	19 (63.3)	16 (61.5)	0.999	21 (61.8)	
Female	11 (36.7)	10 (38.5)		13 (38.2)	0.999
				(36.4)	
Median age, months (IQR)	18.5 (15–32)	92.5 (68–130)	<0.001	32 (18–44)	0.054
Pharyngotonsillitis	29 (96.7)	10 (38.5)	<0.001	23 (67.7)	0.003
Rhinitis	13 (43.3)	0	<0.001	7 (20.6)	0.062
Cough	7 (23.3)	2 (7.7)	0.154	1 (2.9)	0.021
Bronchitis and/or pneumonia	4 (13.3)	5 (19.2)	0.719	0	0.043
Vomiting	8 (26.7)	13 (50)	0.099	4 (11.8)	0.199
Diarrhea	8 (26.7)	11 (42.3)	0.265	9 (26.5)	0.999
Non-exudative conjunctivitis	6 (20)	13 (50)	0.025	27 (79.4)	<0.001
Cervical lymphadenopathy	13 (43.3)	6 (23.1)	0.159	27 (79.4)	0.004
Oral mucositis or cheilitis	1 (3.3)	10 (38.5)	0.001	30 (88.2)	<0.001
Skin rash	3 (10)	13 (50)	0.001	27 (79.4)	<0.001
Diaper/perineal rash	1 (3.3)	0	0.999	7 (20.6)	0.041
Extremity changes	0	7 (26.9)	0.003	24 (70.6)	<0.001
Complete KD	-	-	-	25 (73.5)	-
Length of hospital stay, days	3.5 (2–4)	9 (7–11)	<0.001	8 (7–13)	<0.001
Fever duration, days	5 (4–8)	7 (5–9)	0.112	9 (7–11)	<0.001
Sterile pyuria^a^	4 (14.3)	3 (11.5)	-	7 (21.2)	0.722

*IQR* interquartile range

^a^Information about sterile pyuria was available in 28 out of 30 AI, 13/26 MIS-C, and 33/34 KD patients

**Table 2:** Laboratory findings of patients with upper respiratory tract Adenovirus infection (AI), multisystem inflammatory syndrome in children (MIS-C), and Kawasaki disease (KD)

	**AI** **n = 30**	**MIS-C** **n = 26**	**AI vs MIS-C** **p-value**	**KD** **n = 34**	**AI vs KD** **p-value**
	**Median (IQR)**	**Median (IQR)**		**Median (IQR)**	
CRP, mg/dL	7.5 (5.3–11.9)	17.3 (9.1–21.5)	<0.001	10.1 (5.6–15.7)	0.181
Procalcitonin, ng/mL	1.2 (0.6–2.6)	4.9 (1.8–13.1)	0.004	1.9 (0.8–3.5)	0.456
WBC, *10^9^/L	14.6 (11.2–19.4)	11 (7.1–15.5)	0.052	14.8 (11.6–19.4)	0.946
Neutrophils %	56.4 (50.3–66.7)	83.3 (76.3–86.7)	<0.001	72.6 (60.8–79.9)	<0.001
Monocytes %	1.3 (1.1–1.5)	2.9 (1–4.8)	<0.001	5.6 (4–7.9)	<0.001
Eosinophils %	0.2 (0.1–1.4)	0.5 (0.2–1.2)	0.370	1.3 (0.4–2.2)	0.009
Lymphocytes %	33.5 (24.6–42.7)	12.5 (10.3–19.6)	<0.001	22 (14.5–29.8)	<0.001
Hemoglobin, g/dL	11.2 (10.5–12.3)	10.6 (9.8–10.9)	0.018	11 (10.3–11.4)	0.299
Platelets, *10^9^/L	330 (293–469)	154 (129–288)	<0.001	390 (332–485)	0.156
AST, U/L	37 (32–46)	34 (26–46)	0.409	32 (27–44)	0.137
ALT, U/L	14 (12–19)	24 (19–38)	<0.001	22 (13–46)	0.022
Albumin, g/L	40.9 (39.3–43)	30.5 (27.4–33.6)	<0.001	33.2 (31.3–38.1)	<0.001

* multiplied by

*IQR* interquartile range, *CRP* C-reactive protein, *WBC* white blood cells, *AST* aspartate aminotransferase, *ALT* alanine aminotransferase

Since MIS-C and KD require different management than AI, a multivariable analysis was performed to identify independent predictive factors for MIS-C and KD as compared to AI (Table 3).

**Table 3:** Predictors of multisystem inflammatory syndrome in children (MIS-C) and Kawasaki Disease (KD) as compared to Adenovirus infection (AI)

**Variable**	**AI** **n = 30**	**MIS-C + KD** **n = 60**	**Simple model** **Clinical Score**	**Complex model** **Clinic-lab score**
	**n (%)**	**n (%)**	**OR (95%CI)**	**OR (95%CI)**
Age ≥ 36 months	5 (16.7)	38 (63.3)	11.85 (2.26–61.97)	22.40 (2.72–184.62)
Absence of pharyngotonsillitis, rhinitis, or cough	14 (46.7)	53 (88.3)	21.02 (2.80–157.94)	24.80 (2.20–279.63)
Conjunctivitis and/or oral mucositis or cheilitis	7 (23.3)	47 (78.3)	18.43 (2.66–127.52)	16.63 (2.23–123.84)
Skin rash/extremity changes	3 (10)	45 (75)	14.07 (2.03–97.68)	30.01 (2.15–418.68)
Neutrophils/lymphocytes ratio ≥ 1.3	19 (63.3)	56 (93.3)	-	93.97 (1.89–4660.82)

*CI* confidence interval, *OR* odds ratio

**Fig. 1:**
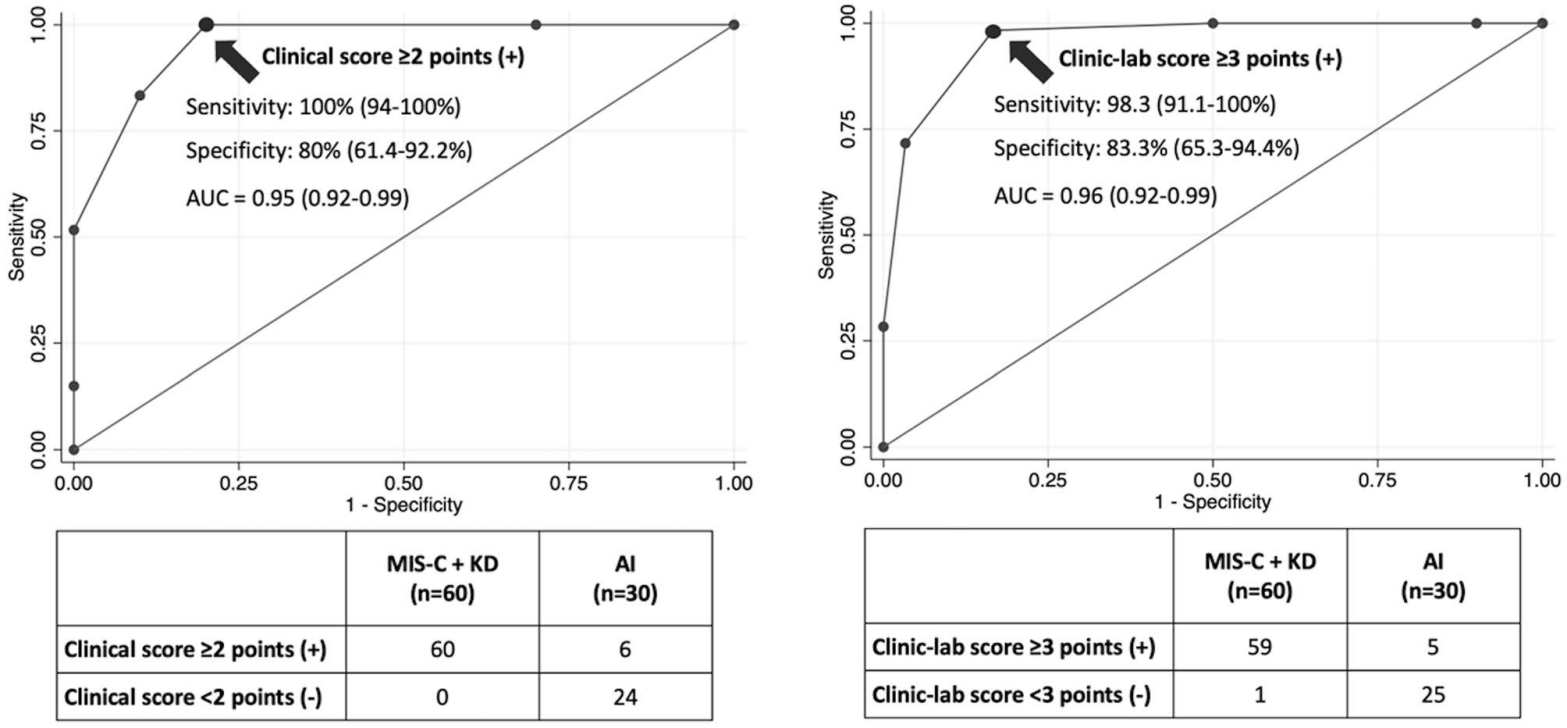
Scoring system for differential diagnosis between AI vs KD or MIS-C pediatric patients. **Left side:** Simple model (“clinical score”) based on 5 clinical parameters: age ≥ 36 months; absence of pharyngotonsillitis, rhinitis or cough; conjunctivitis and/or oral mucositis or cheilitis; skin rash or extremity changes. **Right side:** Complex model ("clinic-lab score”) based on the same 5 clinical parameters and 1 laboratory parameter: neutrophils/lymphocytes ratio

Two scoring models were subsequently constructed to distinguish KD and MIS-C from AI patients. The clinical score, or simple model, was based on 5 clinical parameters: age ≥ 36 months, conjunctivitis and/or oral mucositis or cheilitis, skin rash or extremity changes, and absence of pharyngotonsillitis, rhinitis, or cough. The mixed clinical and laboratory (named “clinic-lab") score, or complex model, included the same clinical criteria and 1 laboratory parameter: the neutrophils/lymphocytes ratio. A score of 1 point was attributed to each predictor of AI, yielding a total score ranging from 0 to 5 points (simple model) and from 0 to 6 points (complex model). According to the ROC analysis, a total score ≥2 for the simple model (Fig. 1a) yielded 100% (CI 94–100%) sensitivity and 80% (CI 61.4–92.2%) specificity for MIS-C and KD diagnosis as compared to AI, with an area under the ROC curve of 0.95 (0.92–0.99). In the complex model (Fig. 1b), a total score ≥3 yielded 98.3% (CI 91.1–100%) sensitivity and 83.3% (CI 65.3–94.4%) specificity for MIS-C and KD, as compared to AI, with an area under the ROC curve of 0.96 (0.92–0.99).

## Discussion

Since children with KD, MIS-C, and upper respiratory tract AI share a similar clinical presentation, the distinction between these diseases may be challenging [10]. However, an accurate differential diagnosis is mandatory, as KD and MIS-C require higher levels of monitoring and treatment than AI. The study findings show that a clinical scoring system, including simple data such as age and clinical signs or symptoms, such as pharyngotonsillitis, rhinitis or cough, conjunctivitis or oral mucositis/cheilitis, and skin rash, may be a useful tool for the differential diagnosis between pediatric patients with KD or MIS-C and those with upper respiratory tract AI.

The development of a clinical score in patients with prolonged high-fever fever and signs and symptoms mimicking KD, MIS-C, and AI could be a simple and cost-effective method to distinguish between patients who need close monitoring and more aggressive treatment and those who might be safely managed at home. Although this scoring system can be a useful tool for both inpatients and outpatients, its utility potentially becomes more pronounced in the case of outpatients, leading to a likely reduction in emergency department admissions for children with a score below 2. In addition, the high sensitivity of this scoring system reduces the risk of missing potential KD and MIS-C cases.

The inclusion of laboratory data in the score helped increase the specificity to 83%, supporting the role of blood tests as an additional tool in the differential diagnosis of these conditions presenting similar clinical features.

To our knowledge, this study is the first attempt to develop such a tool for frontline clinicians to make the differential diagnosis between these diseases before these children get to the emergency department. Some scores have already been developed to early identify KD patients at risk for severe forms of the disease, such as those with coronary involvement and IVIG non-responsiveness [11–15]. Recently, a machine learning model system has shown high accuracy in discriminating KD, MIS-C, and other febrile illnesses in inpatients using clinical features and blood tests [16]. However, these scoring systems do not help in the differential diagnosis of febrile outpatient children. Furthermore, new advanced tests have been developed in recent years to increase diagnostic accuracy in KD and to distinguish it from other febrile conditions, such as those based on gene-expression signature [17, 18]. Although these tests show high sensitivity and specificity for early detection of KD and their use has become wider, their accessibility is still limited.

From a clinical perspective, in our cohort, the signs of mucocutaneous inflammation, such as oral mucositis, cheilitis, conjunctivitis, and skin rash, are significantly more frequent in KD and MIS-C patients than in AI patients. In addition, cervical lymphadenopathy and diaper rash were found to be peculiar in patients with KD compared to the other conditions. Interestingly, a rash located in the diaper area is a clinical sign historically described in KD, despite it not being listed among KD diagnostic criteria [19].

On the other hand, patients with AI were more likely to have respiratory symptoms than KD and MIS-C children; pharyngotonsillitis was the most distinctive clinical sign of AI, consistently with the respiratory tract infection.

Fever duration was longer in KD than in AI and MIS-C patients. A possible reason for the shorter fever duration in MIS-C compared to KD might be an earlier treatment thanks to increased awareness of this entity during the pandemic and, consequently, a quicker diagnosis.

Surprisingly, laboratory data only slightly increased the specificity for the diagnosis of MIS-C or KD in a complex model that included the neutrophils/lymphocytes ratio. However, it can contribute to the diagnostic process as an additional tool, and it is a main pillar of the algorithm for incomplete KD along with the evidence of coronary involvement. In our cohort, neutrophil and monocyte counts were higher in KD and MIS-C than AI patients, while the lymphocyte count was higher in AI children, as expected in viral infections; inflammatory markers were higher in MIS-C than AI, suggesting a more intense inflammation. The timing of IVIG administration is crucial for reducing the incidence of coronary lesions [1]: an earlier diagnosis from AI can lead to an earlier treatment. Similarly, in MIS-C patients, early administration of treatment seems to be beneficial, preventing the progression of the inflammatory process and reducing the risk of admission to the ICU [20].

Finally, the identification of AI in patients with URTI enables guidance for proper treatment, reduces hospital costs, and minimizes the excessive use of antibiotics. However, although in most cases Adenovirus causes relatively mild, self-limiting infections as in all of our patients, severe clinical pictures have also been reported, ranging from pneumonia-induced acute respiratory failure to myocarditis, encephalitis, encephalomyelitis, and aseptic meningitis [7]. Moreover, two pediatric cases of Adenovirus-induced reactive infectious mucocutaneous eruption have been recently described [21]. These patients showed severe, erosive mucositis and persistent fever, with features overlapping with both KD and the Steven-Johnson Syndrome/Toxic Epidermal Necrolysis spectrum; although the final diagnosis was Adenoviral-induced rash and mucositis, they required an immunomodulation therapy with IVIG and systemic corticosteroids. It is also well-known that patients with AI may develop KD, as in one of our cases [1]. In all these situations, treatment should be based on the clinical severity of the patients and not only on the specific diagnosis.

Our study presents several limitations. First, the relatively small sample size, partly due to the low incidence of MIS-C and KD may have affected the confidence in our estimates, as revealed also by the wide confidence intervals of the ORs for the predictors included in the models: nevertheless, at post-hoc analysis considering that the simple and the complex model increased the probability of MIS-C and KD from 66 to 90% and 92% respectively, we computed a power of 0.86 and 0.91 respectively, yielding a fair degree of confidence in our results. The duration of enrolment and the monocentric nature of the study are limitations of the present study as well. It is crucial to highlight that our scores have not been applied to a validation cohort and should not be considered until an external validation has been completed. Validation of a score in a separate, independent cohort is a measure of its reliability, and a scoring system should only be used if it has been thoroughly validated [22].

## Conclusion

To our knowledge, this is the first scoring system attempting to provide differential diagnosis between KD, MIS-C, and AI before persistently febrile children get to the emergency department. One of the practical implications of the presented score is a contribution to a more effective use of resources, allowing hospitals to efficiently provide patients with a higher need for assistance and limiting public healthcare spending. In children with AI, the risk of overtesting is remarkable, leading to the prescription of additional blood tests, microbiological investigations, and cardiac evaluations. Furthermore, the score could help the clinician not underestimate the true relative risk of KD or MIS-C among patients with an incidental Adenovirus detection on upper respiratory tract specimens. However, external validation of the score in an independent cohort is necessary to confirm its reliability and allow its use in the clinical practice.

## Data Availability

The data presented in the study are available from the corresponding author upon reasonable request.

## List of abbreviations

AHA: American Heart Association
AI: Adenoviral infections
ALT: Alanine aminotransferase
AST: Aspartate aminotransferase
CI: Confidence intervals
CRP: C-reactive protein
Hb: Hemoglobin
ICU: Intensive Care Unit
IVIG: Intravenous Immunoglobulins
KD: Kawasaki Disease
MIS-C: Multisystem Inflammatory Syndrome in Children
NPV: Negative predictive value
ORs: Odds ratios
PLT: Platelets
PPV: Positive predictive value
ROC: Receiver operating characteristic
WBC: White blood cell count
